# Novel PI3K/AKT targeting anti-angiogenic activities of 4-vinylphenol, a new therapeutic potential of a well-known styrene metabolite

**DOI:** 10.1038/srep11149

**Published:** 2015-06-08

**Authors:** Grace Gar-Lee Yue, Julia Kin-Ming Lee, Hin-Fai Kwok, Ling Cheng, Eric Chun-Wai Wong, Lei Jiang, Hua Yu, Hoi-Wing Leung, Yuk-Lau Wong, Ping-Chung Leung, Kwok-Pui Fung, Clara Bik-San Lau

**Affiliations:** 1 Institute of Chinese Medicine, The Chinese University of Hong Kong, Shatin, New Territories, Hong Kong; 2 State Key Laboratory of Phytochemistry and Plant Resources in West China (CUHK), The Chinese University of Hong Kong, Shatin, New Territories, Hong Kong; 3 School of Biomedical Sciences, The Chinese University of Hong Kong, Shatin, New Territories, Hong Kong

## Abstract

The pneumo- and hepato-toxicity of 4-vinylphenol (4VP), a styrene metabolite, has been previously reported. Nevertheless, the present study reported the novel anti-angiogenic activities of 4VP which was firstly isolated from the aqueous extract of a Chinese medicinal herb *Hedyotis diffusa*. Our results showed that 4VP at non-toxic dose effectively suppressed migration, tube formation, adhesion to extracellular matrix proteins, as well as protein and mRNA expressions of metalloproteinase-2 of human endothelial cells (HUVEC and HMEC-1). Investigation of the signal transduction revealed that 4VP down-regulated PI3K/AKT and p38 MAPK. Besides, 4VP interfered with the phosphorylation of ERK1/2, the translocation and expression of NFkappaB. In zebrafish embryo model, the new blood vessel growth was significantly blocked by 4VP (6.25–12.5 μg/mL medium). The VEGF-induced blood vessel formation in Matrigel plugs in C57BL/6 mice was suppressed by 4VP (20–100 μg/mL matrigel). In addition, the blood vessel number and tumor size were reduced by intraperitoneal 4VP (0.2–2 mg/kg) in 4T1 breast tumor-bearing BALB/c mice, with doxorubicin as positive control. Together, the *in vitro* and *in vivo* anti-angiogenic activities of 4VP were demonstrated for the first time. These findings suggest that 4VP has great potential to be further developed as an anti-angiogenic agent.

Angiogenesis is essential for tumor growth and it is believed that blocking angiogenesis could be a strategy to arrest tumor growth and metastasis^1^. In the last decades, several drugs which target the tumor vasculature and inhibit tumor angiogenesis have been discovered^2^ and some antiangiogenic agents are approved for clinical use, such as humanized anti-VEGF-A antibody bevacizumab, tyrosine kinase inhibitors sorafenib and sunitinib^3^. Hundreds of late-stage clinical trials on these agents are currently in progress^4^,^5^. Antiangiogenic therapies, which are aimed at suppressing new blood vessel growth, have the potential to become a new target focus or a major adjuvant for cancer treatment. Many chemopreventive molecules isolated from natural products, including taxol^6^, epigallocatechin gallate^7^, curcumin^8^, farnesiferol C^9^, and cyclopeptide RA-V^10^ are also known to inhibit angiogenesis. Nevertheless, there is a continuing need for new antiangiogenic drugs, especially from natural products including Chinese herbs. In the present study, we demonstrated the antiangiogenic activities of a simple and common compound, 4-vinylphenol, which was firstly found in Chinese folk medicinal herb, *Hedyotis diffusa* Willd.

The herb *Hedyotis diffusa* (HD) is the dried whole plant of *Hedyotis diffusa* Willd. (family Rubiaceae). The plant is widely distributed in Northeast Asia and used as anti-cancer herb in folk remedies in Hong Kong, Taiwan and Southern China^11^,^12^. HD was shown to contain anthraquinones, terpenoids, steroids, flavonoids, organic acid, and polysaccharides^13^. Previous pharmacological studies reported that the water extract^11^,^12^,^14^,^15^, ethanolic extract^16^,^17^ of the herb as well as ursolic acid^18^,^19^ isolated from the herb possessed anti-proliferative activities in cancer cells or antitumor activities in tumor-bearing animals. On the other hand, the anti-angiogenic activities of *Hedyotis diffusa* ethanolic extract have been demonstrated recently. Lin *et al.* showed that the ethanolic extract of HD inhibited the proliferation and tube formation of HUVEC and the expressions of VEGF-A and VEGFR2^16^. However, another study suggested that the main components (including two iridoid glucosides and ursolic acid) in the methanol extract of the herb had almost no effect on zebrafish angiogenic vessel formation^20^. In our pilot studies, the hot aqueous extract of HD could inhibit the proliferation, tube formation, migration of HUVEC as well as blood vessels formation in zebrafish (Fig. 1C, D). Hence, we aimed to isolate the active component(s) responsible for the anti-angiogenic effects of HD aqeuous extract using bioassay-guided fractionation. As a result, an active compound was isolated and identified as 4-vinylphenol (4VP, Fig. 1A).

4VP naturally occur in coffee, peanuts and wild rice^21^,^22^. 4VP could also be isolated from the dried root of *Vetiveria zizanioides* (Poaceae family)^23^ and *Asplenium trichomanes* (Aspleniaceae family)^24^. It was also found to be the metabolite of *p*-coumaric and ferulic acid by lactic acid bacteria in wine^25^. 4VP has been identified as a metabolite of styrene and the pneumotoxicity and hepatotoxicity of 4VP were previously documented a decade ago^22^,^26^,^27^. Nevertheless, the biological activity of 4VP has not been evaluated. Since 4VP was isolated from HD using anti-angiogenic bioassay-guided fractionation, we aimed to investigate the underlying mechanism of action of 4VP *in vitro* and *in vivo*. Here we reported for the first time that 4VP possessed anti-angiogenic effects in both HUVEC and HMEC-1 human endothelial cell lines and the PI3K/AKT, ERK and p38 signaling pathway was involved in 4VP’s activities. Besides, the non-toxic dose of 4VP could reduce tumor size and blood vessel growth in tumors in breast tumor-bearing mouse model.

## Materials and Methods

### Chemicals and reagents

Dried whole plants of *Hedyotis diffusa* Willd. (HD) were purchased from herbal suppliers in Hong Kong. Organoleptic and chemical authentication was accomplished in accordance with the reference books^28^,^29^ as described in our previous study^30^. Authenticated voucher specimen was deposited in the museum of Institute of Chinese Medicine, the Chinese University of Hong Kong, with voucher specimen number 20073131. 4-vinylphenol (4VP) was initially isolated from HD aqueous extract and subsequently purchased from commercially available source (10% w/v solution in propylene glycol, Alfa Aesar, UK) for the *in vitro* and *in vivo* studies.

The human umbilical vein endothelial cells (HUVEC), human microvascular endothelial cells (HMEC-1) and mouse breast tumor cells (4T1) were purchased from American Type Culture Collection (MD, USA). Dulbecco’s modified Eagle’s medium/ F12 (DMEM/F12), DMEM medium, RPMI medium, fetal bovine serum (FBS), penicillin-streptomycin, trypsin-EDTA, recombinant vascular endothelial growth factor (VEGF), Trizol, SuperScript III Reverse Transcriptase, dNTP were obtained from Life Technologies (NY, USA). MCDB 131 medium, endothelial cell growth supplement, epidermal growth factor, basic human fibroblast growth factor (bFGF), hydrocortisone, heparin, gelatin, hematoxylin & eosin, 3-(4,5-dimethylthiazol-2-yl)-2,5-diphenyl-tetrazolium bromide (MTT), SU5416, doxorubicin and Drabkin’s reagent were obtained from Sigma (MO, USA). Basement membrane matrix Matrigel (Growth factor reduced) was from BD Biosciences (NJ, USA). The sources and catalogue numbers of the antibodies for Western blot were listed in Supplementary Information Table S1. [Methyl-^3^H]-thymidine and unifilters were obtained from PerkinElmer (MA, USA). Real-time PCR reagent iTaq Fast SYBR Green Supermix was obtained from Bio-Rad (Hong Kong). Transwell polycarbonate cell culture inserts (6.5 mm diameter, 8 μm pore size) were from Costar (MA, USA).

### Extraction and isolation of 4VP

The dried herb *Hedyotis diffusa* was extracted twice with distilled water under reflux for 1 hour. The purified compound (4-vinylphenol) was isolated from the aqueous extract by a series of column chromatography. The identification of the 4-vinylphenol was based on the ^1^H and ^13^C NMR spectral analysis and mass spectrometry. The content of 4-vinylphenol in HD aqueous extract was determined by HPLC analysis. The detailed procedures of isolation and quantification were described in Supplementary Information.

### Cell culture

The HUVEC were maintained in DMEM/F12 medium containing 100 μg/mL heparin and 30 μg/mL endothelial cell growth supplement. The HMEC-1 were maintained in MCDB 131 medium containing 2 mM glutamine, 1 μg/mL hydrocortisone and 10 ng/mL epidermal growth factor. The mouse breast tumor cells (4T1) were maintained in RPMI. All of the media were supplemented with 10% v/v heat-inactivated FBS, 100 units/mL penicillin-streptomycin. The cells were incubated at 37 °C in a humidified atmosphere of 5% CO_2_. When the cells reached 80% confluence in culture flasks, trypsin-EDTA was used to remove the cells and the cells were used in experiments or reseeded in flask. 4VP (10% w/v solution in propylene glycol) was diluted 10X by preparing at 10 mg/mL (83.2 mM) in dimethylsulfoxide (DMSO) and stored at −20 °C and reconstituted in appropriate media prior to the experiments. The vehicle control cultures received the vehicle solvent (0.45% v/v DMSO and 0.05% propylene glycol).

### Cell proliferation and cytotoxicity assay

The HUVEC or HMEC-1 (3 × 10^4^/mL) were seeded in 96-well flat-bottom culture plates with 100 μL culture medium and incubated overnight. Subsequently, 100 μL culture media containing various concentrations (6.25–50 μg/mL) of 4VP were added into the wells. Then the plates were incubated at 37 °C for 48 hours. Plain medium containing vehicle solvent were added to the control wells. The effects of 4VP on the proliferation and viabilities of HUVEC and HMEC-1 were assessed by thymidine incorporation and MTT assays, respectively, as described in our previous studies^31^.

Tube formation matrigel-based assay, modified Boyden chamber assay and scratch wound assay have been performed to evaluate the ability to form capillary tube-like structures, migration and motility of the cells, respectively. The procedures of these assays were described in Supplementary Information.

### Zebrafish maintenance, embryo collection and treatment

Transgenic zebrafish Tg(*fli1*:EGFP)y1, in which the endothelial cells express enhanced Green Fluorescent Proteins (eGFP), was obtained from Zebrafish International Resource Center (ZIRC) and maintained as described previously^32^,^33^,^34^. Healthy, limpid, and regular embryos were collected at their 1–4 cell stage and transferred into a 24-well microplate, with 20 embryos per well. The medium was replaced by medium containing different concentrations of 4VP. A maximum of 0.45% v/v DMSO and 0.05% propylene glycol was used as vehicle control group. Positive control SU5416 (2 μM) was added. After 24 hours of treatment, the viability and gross morphological state of embryos were examined. At 48 hours post-fertilization (hpf), the intersegmental vessels were observed in SU5416- and 4VP-treated embryos. At 72 hpf, photos of the sub-intestinal vessels (SIV) of the embryos were taken under a fluorescent microscope (Olympus IX71) by a digital camera (Diagnostic Instruments, Inc., USA) at 100X magnification. The length of the SIV was measured with the software ImageJ^32^.

### Extracellular matrix cell adhesion assay and gelatin zymography

Extracellular matrix cell adhesion assay and gelatin zymography have been performed to assess the effects of 4VP on cell adhesion and matrix metalloproteinases activities, respectively. The procedures of these assays were described in Supplementary Information.

### NFκB p65 transcription factor assay

To assess NFκB activation by 4VP, the nuclear fractions of 4VP-treated endothelial cells were isolated and the bound NFκB was detected using NFκB p65 Transcription Factor Assay Kit (ab133112, abcam, UK). The assay was carried out according to the procedures recommended in the assay kit manual.

### Western blot analysis

Human endothelial cells HUVEC and HMEC-1 (1 × 10^6^/mL) were seeded and incubated for 24 hours to allow attachment. Different concentrations (20 or 40 μg/mL) of 4VP were added to the dishes and incubated for 24 or 48 hours. For the measurement of NF-κB activation, endothelial cells were preincubated with 20 or 40 μg/mL of 4VP for 6 hours and treated with 15 ng/mL TNF-α for 1 hour. After treatments, cells were collected, washed and lysed as described previously^10^,^32^.

### Real time-PCR analysis

Human endothelial cells HUVEC and HMEC-1 (1 × 10^6^/mL) were seeded and incubated for 24 hours. Different concentrations (20 or 40 μg/mL) of 4VP were added to the dishes and incubated for 24 or 48 hours. After treatments, cells were harvested and washed. Total RNA was extracted and quantitated as described previously^10^,^32^. The details of the real time semi-quantitative PCR and the sequences of the primers are listed in the Supplementary Information and Table S2.

### *In vivo* Matrigel plug assay

Male C57BL/6 mice (6 weeks old) were supplied and maintained by Laboratory Animal Service Center, the Chinese University of Hong Kong. Matrigel (500 μL) was mixed with heparin (10 U/mL), VEGF 100 ng/mL and 4VP (20 or 100 μg/mL) prior to subcutaneous injections into the flanks of mice. Naive controls were obtained by injecting mice with matrigel in the absence of VEGF and 4VP. After 7 days, the matrigel plugs were removed and photographed. The hemoglobin content of the matrigel plugs was quantified using Drabkin’s reagent kit (Sigma, USA). Hemoglobin content was expressed as mg/mg of wet matrigel plug^10^.

### Mouse mammary tumor model

Female BALB/c mice (6–8 weeks old) were supplied and maintained by Laboratory Animal Services Center. Mouse mammary tumor cells 4T1 (4 × 10^5^) resuspended in 0.2 mL PBS, were subcutaneously inoculated at the mammary fat pad of each mouse. Treatments were initiated 8 days after tumor cell implantation and lasted for 4 weeks. After 4T1 cell implantation, the tumor-bearing mice were randomly assigned into 4 groups (n = 15): vehicle control group, doxorubicin group (0.5 mg/kg, once a week for 4 weeks), 4VP-L group (0.2 mg/kg) and 4VP-H group (2 mg/kg). The doses of 4VP for mice were calculated using the human equivalent daily dose of HD^29^ and the conversion factor for mice published by Food and Drug Administration, USA. The 4VP was diluted in PBS and administered intraperitoneally to mice daily. The vehicle control group received the vehicle solvent (0.45% v/v DMSO and 0.05% propylene glycol) in PBS. During 4VP treatment, the body weight of each mouse was measured once a week during treatment period. At the end of treatment, mice were sacrificed and the lungs and livers were removed for quantification of tumor burden. Tumors of mice from different groups were removed for histological analysis.

All experimental methods in zebrafish and mice were carried out in accordance with the approved guidelines specified by the Animal Experimentation Ethics Committee of the Chinese University of Hong Kong (CUHK). All experimental protocols were approved by the Animal Experimentation Ethics Committee of CUHK with reference numbers Ref No. 10/013/MIS and 10/051/MIS.

### Histological and immunohistochemical analysis

Tumors, lungs and livers were fixed in 10% buffered formalin for 10 days at room temperature. Then samples were paraffin embedded, sectioned longitudinally at 5 μm. The level of cell apoptosis in tumor sections was determined with TUNEL assay using *in situ* cell death POD kit (Roche, Germany). The assay was carried out according to the procedures recommended in the assay kit manual. The stained tumor sections were examined and photographed. Four fields of tumor sections were randomly selected, and the area of apoptotic cells was calculated for each field. The tumor sections were stained with anti-mouse CD31 (Dianova, Germany) antibody using an immunohistochemical method and the details were described in Supplementary Information.

The sections of lung and liver were stained with hematoxylin & eosin and examined and photographed as described previously^35^. Tumor burden, defined as the tumor area, was calculated from the section of the lung or liver and expressed as an average percentage of tumor area to lung or liver area in each treatment group.

### Statistical analysis

Data were expressed as mean + SD (*in vitro*) or mean + SEM (*in vivo*). Statistical analyses and significance were analyzed by one-way ANOVA with Tukey’s post-hoc test using GraphPad PRISM software version 5.0 (GraphPad Software, USA). In all comparisons, *p* < 0.05 was considered statistically significant.

## Results

### Structure elucidation of 4VP

**Figure 1:**
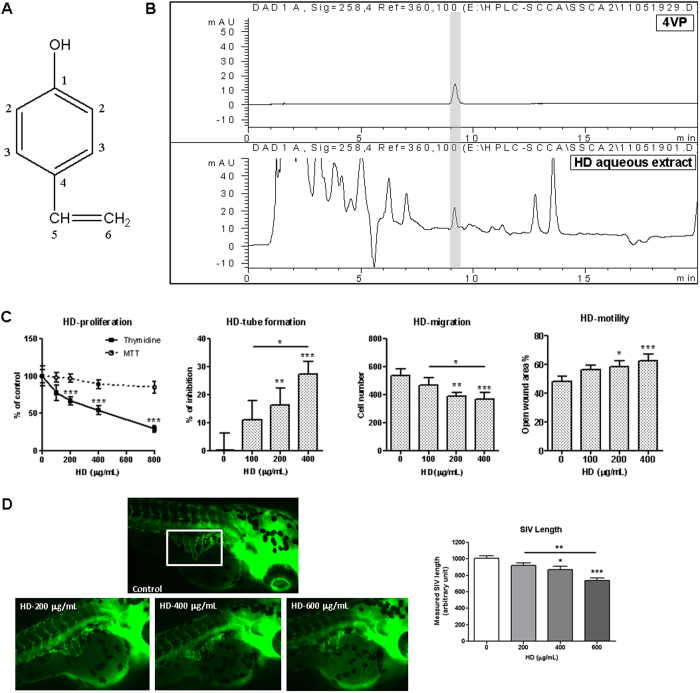
(**A**) Chemical structure of 4-vinylphenol (4VP). (**B**) HPLC chromatograms of 4VP and HD aqueous extract. (**C**) HD aqueous extract inhibited cell proliferation, tube formation, migration and motility in HUVEC in a concentration-dependent manner. Results are expressed as the mean + SD of 3–4 independent experiments. (**D**) HD aqueous extract inhibited subintestinal vessel formation in zebrafish embryos. Results are expressed as the mean + SEM (n = 30–40) of 3 independent experiments. Differences between the treated and untreated control groups were determined by one-way ANOVA with Tukey’s post-hoc test. *p < 0.05, **p < 0.01, ***p < 0.005 as compared among groups.

The structure of 4VP was elucidated by ^1^H and ^13^C NMR spectroscopic analysis and the spectral data were listed in Table S3, which were in accordance with the reported data in the literature^36^. The chemical structure of 4VP was shown in Fig. 1A. The content of 4VP in HD aqueous extract was determined to be 0.037 ± 0.003% with the HPLC method as described above. The HPLC chromatograms of 4VP and HD aqueous extract were shown in Fig. 1B.

### Anti-angiogenic activities of HD aqueous extract

Our results of pilot studies showed that HD aqueous extract (HD) at 200 and 400 μg/mL significantly inhibited HUVEC cell proliferation, tube formation, migration and motility, without significant cytotoxic effect (assessed by MTT assay) (Fig. 1C). Besides, the *in vivo* anti-angiogenic activities of HD were also demonstrated in zebrafish model. Zebrafish embryos treated with HD at 400 or 600 μg/mL significantly inhibited subintestinal vessel formation by 14% and 27%, respectively in the embryos (Fig. 1D).

The HD was further partitioned and fractionated. The active components in HD responsible for the HUVEC cell proliferation inhibition were isolated using *in vitro* bioassay-guided fractionation method. The findings suggested that within the active fraction F1-2A, 4VP was responsible for the inhibitory activities in endothelial cells. Hence, the underlying mechanisms of action of 4-VP have been investigated.

### Inhibitory effects on cell proliferation and cytotoxicity of 4VP in endothelial cells

**Figure 2:**
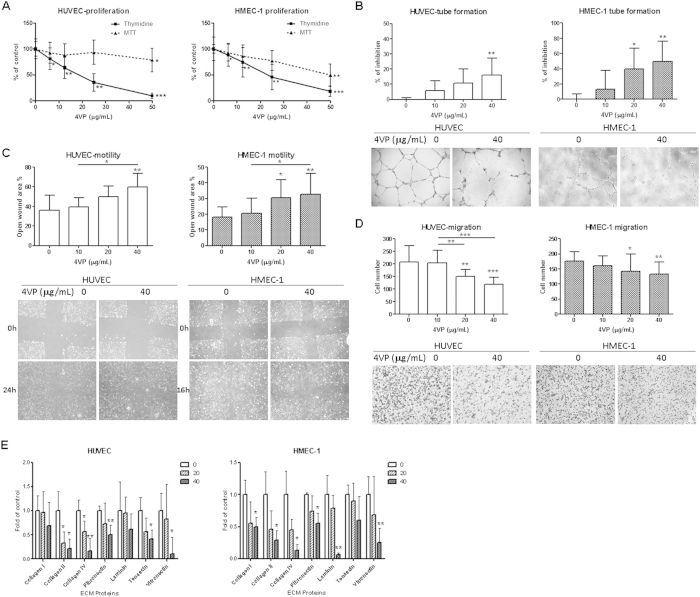
Effects of 4VP on cell proliferation, tube formation, migration, motility and cell adhesion to ECM proteins of HUVEC and HMEC-1. (**A**) Cells were treated with increasing concentrations of 4VP for 48 hours, and cell proliferation was determined by the [methyl-^3^H] thymidine incorporation. Results are expressed as the mean % ratio of count per minute in treated and vehicle-treated control cells (mean ± SD of 4 independent experiments with 5 wells each). (**B**) Quantification of tube formation in HUVEC and HMEC-1 were shown. Representative photomicrographs showing the tube structures of HUVEC or HMEC-1 following 8 or 6 hours treatments, respectively, with vehicle (0 μg/mL) or indicated concentrations of 4VP. (**C**) Quantification of cell migration of HUVEC and HMEC-1 in Boyden chambers were shown. Representative photomicrographs showing the migrated and stained cells on the lower side of membranes. (**D**) Quantification of wound-induced cell motility in HUVEC and HMEC-1 were shown. Representative photomicrographs showing the cells migrated across the scratch wound in the presence or absence of 4VP after 16 or 24 hours incubation. (**E**) Effects of 4VP on cell adhesion to extracellular matrix proteins. Cells treated with 4VP (20 or 40 μg/mL) were added to a precoated plate containing different ECM proteins. The adhered cells were quantitated and results were shown. Results are expressed as the mean percentage of control (mean + SD of 3 independent experiments). Differences between the treated and vehicle-treated control groups were determined by one-way ANOVA with Tukey’s post-hoc test. Differences among treated groups were determined by one-way ANOVA with Tukey’s post-hoc test (**B**, **C** and **D**). *p < 0.05, **p < 0.01, ***p < 0.005 as compared among groups.

Different concentrations of 4VP were tested in HUVEC and HMEC-1 to examine its effect on the cell proliferation. As shown in Fig. 2, 4VP (6.25–50 μg/mL) was found to significantly inhibit the proliferation of HUVEC and HMEC-1 in a concentration-dependent manner after 48 hours treatment (Fig. 2A). The concentrations producing 50% growth inhibition (IC_50_) of 4VP on HUVEC and HMEC-1 were 15.31 and 21.43 μg/mL, respectively. The presence of 4VP vehicle, propylene glycol (up to 0.5% v/v), did not affect the cell proliferation of endothelial cells (data not shown).Besides, the cytotoxicity of 4VP in both endothelial cell lines (HUVEC and HMEC-1) was assessed by MTT assay after 48 hours incubation. The concentrations of 4VP producing 50% cell death in HUVEC and HMEC-1 were 100.72 and 52.60 μg/mL, respectively. Low concentration (6.25–25 μg/mL) could cause significant inhibition of proliferation but not cytotoxic effects in both HUVEC and HMEC-1 (Fig. 2A).

### 4VP inhibited tube formation, cell migration and motility in endothelial cells

Endothelial cells when seeded on three-dimensional matrix, such as Matrigel are able to form capillary-like structure. Figure 2B showed the capillary-like tube formation following 8 or 6 hours treatments of HUVEC or HMEC-1 with vehicle (0 μg/mL) or different concentrations of 4VP, respectively. The tube structures were visible in the vehicle control culture well coated with Matrigel. The 4VP treatment (40 μg/mL) significantly inhibited the capillary-like tube formation of HUVEC and HMEC-1 (Fig. 2B, *p* < 0.01).

The effects of 4VP treatment on cell migration by endothelial cells were evaluated using a modified Boyden chamber assay. In vehicle-treated (0 μg/mL) control, lots of endothelial cells migrated from the upper to lower chamber through the membrane after 6 hours incubation, when the lower chamber contained culture medium supplemented with 10% v/v FBS as a chemoattractant. As shown in Fig. 2C, 4VP (20 and 40 μg/mL) blocked the migration of HUVEC and HMEC-1 in a concentration-dependent manner.

The effects of 4VP treatment on cell motility were also evaluated using scratch wound assay (Fig. 2D). Treatments of HUVEC and HMEC-1 with 4VP decreased cell motility compared with vehicle-treated cells in a concentration dependent manner. The open wound areas in 40 μg/mL 4VP-treated wells were significantly greater than those of vehicle-treated wells (*p* < 0.01).

### 4VP decreased endothelial cells adhesion to extracellular matrix (ECM) proteins

The cell adhesion of endothelial cells to different ECM proteins (collagen I, collagen II, collagen IV, fibronectin, laminin, tenascin and vitronectin) was assessed and the results showed that the cell adhesion to several matrix proteins decreased by 4VP treatment. The decreased adhesions observed were statistically significant to collagen II, collagen IV, fibronectin and vitronectin in both cell lines (*p* < 0.05, Fig. 2E). Co-culture of HUVEC with 4VP (40 μg/mL) resulted in 78%, 83%, 49% and 90% reduction of collagen II, collagen IV, fibronectin and vitronectin adhesion, respectively. Besides, the adhesion to all tested ECM proteins of HMEC-1 decreased by 40–94% after 4VP (40 μg/mL) treatment, with laminin decreased the most (94%).

### 4VP inhibited MMP-2 enzyme activity, protein and mRNA expressions in endothelial cells

**Figure 3:**
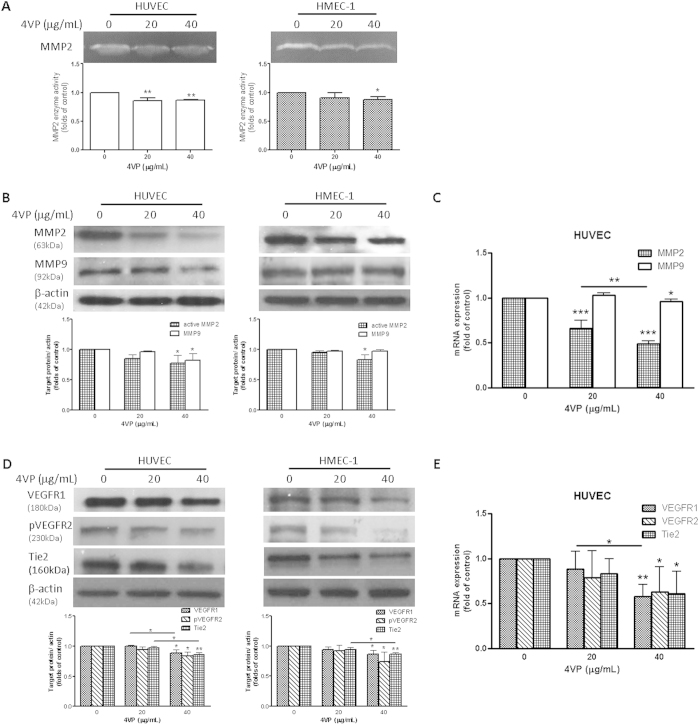
Effects of 4VP on MMPs activities, expressions and VEGFRs expressions in HUVEC and HMEC-1 cells. (**A**) Cells were treated with 4VP (20 or 40 μg/mL) for 24 hours and the MMP activities were determined by zymography. Representative zymograms were shown on HUVEC and HMEC-1 cells. The histograms showed the quantified results of enzyme activities. Western blot analyses of effects of 4VP on (**B**) MMPs and (**D**) VEGFRs expressions. Cells were treated with 4VP (20 or 40 μg/mL) for 24 or 48 hours. Immunoblotting was performed 3–4 times using independently prepared cell lysates and the representative blots were shown. The histograms showed the quantified results of protein levels, which were adjusted with corresponding β-actin protein levels. Quantitative RT-PCR analyses of (C) MMPs and (**E**) VEGFRs gene mRNA. Cells were treated with 4VP (20 or 40 μg/mL) for 24 hours. Data were normalized to corresponding GAPDH expressions in control cells. Proteins and mRNA expressions results are expressed as fold of control (mean fold of control + SD from 3–4 independent experiments). Differences between the treated and vehicle-treated control groups were determined by one-way ANOVA with Tukey’s post-hoc test. *p < 05, **p < 0.01, ***p < 0.005 as compared among groups.

Degradation of the extracellular matrix (ECM) and components of the basement membrane by MMPs play a critical role in tumor invasion and metastasis. Gelatin zymography was carried out to evaluate the effect of 4VP on the activity of MMP-2. As shown in Fig. 3A, MMP-2 in HUVEC and HMEC-1 cell culture supernatant were detected in the gel at molecular weight of 72 kDa. The enzyme activity of MMP-2 was suppressed dose-dependently. Furthermore, the protein expressions of MMP2 were found to be significantly decreased in 4VP-treated HUVEC and HMEC-1 cells (*p* < 0.05, Fig. 3B). On the other hand, the activities of MMP9 in 4VP-treated endothelial cells has also been evaluated using zymography; however, due to the low content of active MMP-9 in both endothelial cell lines, the activities in terms of the digested bands were hardly detected (data not shown). Nevertheless, the protein expressions of MMP9 have been determined in 4VP-treated endothelial cells. The MMP9 expressions were significantly decreased in HUVEC after 4VP treatment while there was no change in HMEC-1 cells (Fig. 3B). As the inhibitory activities of 4VP were stronger in HUVEC, the mRNA expressions of MMP2 and MMP9 in 4VP-treated HUVEC were examined by real-time PCR analysis. As shown in Fig. 3C, the mRNA expression of MMP2 was down-regulated in a concentration-dependent manner after 4VP treatment (*p* < 0.005), while the MMP9 expression was slightly down-regulated by 4VP at 40 μg/mL.

### 4VP decreased the protein and mRNA expressions of VEGFR1, VEGFR2 and Tie 2 in endothelial cells

The effects of 4VP on the expressions of VEGF receptors and tyrosine kinase receptor (VEGF-R1, VEGF-R2 and Tie 2) were examined since binding of VEGF to its receptors is known to initiate angiogenesis. As shown in Fig. 3D, 4VP at 40 μg/mL significantly decreased the expressions of VEGFR1, pVEGFR2 and Tie 2 in both HUVEC and HMEC-1. The effects of 4VP on the mRNA expressions of such receptors have also been evaluated in HUVEC, which do not produce endogenous VEGF as HMEC-1 do^37^. Figure 3E showed that the mRNA expressions of VEGF-R1, VEGF-R2 and Tie 2, which are the receptors of the angiogenic molecules, were down-regulated by 4VP in a concentration dependent manner. The expression of VEGFR1 mRNA was suppressed the most (46%) by 4VP at 40 μg/mL (*p* < 0.005).

### 4VP decreased the protein levels of cyclin B1, cyclin D1 and reduced the phosphorylation of ERK in endothelial cells

**Figure 4:**
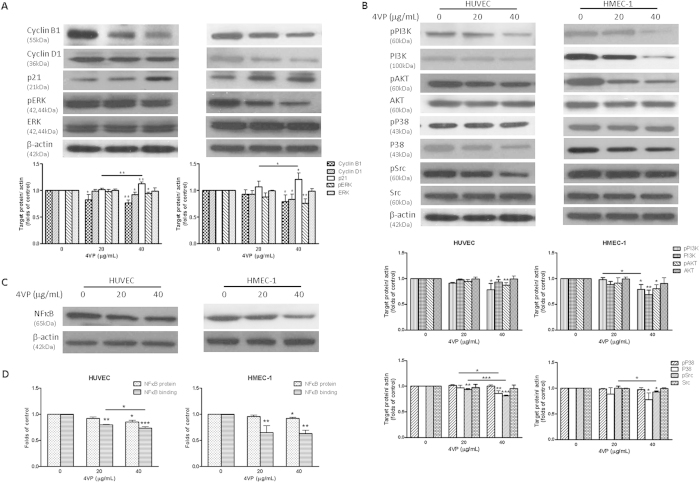
Effects of 4VP on cyclins and signaling kinases expressions and the activation of NFκB transcription factor in endothelial cells. Western blot analyses of effect of 4VP on (**A**) cyclin B1, cyclin D1, p21 and ERK as well as (**B**) PI3K, AKT and P38 MAPK expressions. Cells were treated with 4VP (20 or 40 μg/mL) for 24 or 48 hours. Immunoblotting was performed 3–4 times using independently prepared cell lysates and the representative blots were shown. The histograms showed the quantified results of protein levels, which were adjusted with corresponding β-actin protein levels and expressed as fold of control (mean fold of control + SD from 3–4 independent experiments). Differences between the treated and vehicle-treated control groups were determined by one-way ANOVA with Tukey’s post-hoc test. *p < 0.05, **p < 0.01, ***p < 0.005 as compared among groups. (**C**) Western blot analyses of NFκB expressions in whole cell extract of TNF-α-activated HUVEC and HMEC-1 with or without 4VP. (**D**) Cells were treated with 4VP (20 or 40 μg/mL) for 6 hours and activated with TNF-α (15 ng/mL). The nuclear extracts were assayed for NFκB activation by NFκB p65 Transcription Factor Assay. Data are representative of two independent experiments.

As mentioned in previous section, 4VP inhibited cell proliferation in endothelial cells. Western blot analyses were performed in HUVEC and HMEC-1 to determine whether 4VP was able to inhibit cyclins production. The results showed that 4VP (40 μg/mL) significantly decreased the production of cyclin B1 and cyclin D1 after 24-hours treatment in HUVEC and HMEC-1 (Fig. 4A). In addition, the p21 protein level was significantly increased in 4VP treated both HMEC-1 and HUVEC. Furthermore, to evaluate the effects of 4VP on intracellular signal transduction, the phosphorylation level of extracellular signal-regulated kinase 1/2 (ERK) and were examined in both endothelial cell lines. The results showed that 4VP inhibited the phosphorylation of ERK in a dose-dependent manner in HMEC-1, while 4VP (40 μg/mL) slightly suppressed the expression of pERK in HUVEC (Fig. 4A).

### 4VP downregulated PI3K/AKT and p38 MAP kinase signaling pathways and suppressed NF-κB binding activity

As 4VP was shown to inhibit endothelial cell migration, the signaling pathways have been investigated. 4VP (40 μg/mL) significantly reduced the phosphorylation of PI3K and AKT in both HUVEC and HMEC-1 (*p* < 0.05, Fig. 4B). The levels of total PI3K were reduced in 4VP-treated HMEC-1 in a concentration-dependent manner. Besides, treatment with 4VP significantly reduced the P38 MAP kinase and pSrc in both endothelial cell lines (Fig. 4B), and subsequent inhibited cell migration (Fig. 2C, D).

Upon multiple stimuli, NFκB may enter into nucleus to activate its target genes. To evaluate the effects of 4VP on NF-κB signal transduction, NFκB protein expression and activation in endothelial cells were evaluated by Western blot and NFκB p65 Transcription Factor Assay (abcam), respectively. As shown in Fig. 4C, NFκB protein expressions in TNF-α-activated HUVEC and HMEC-1 were markedly decreased after 4VP treatment for 6 hours. 4VP inhibited TNF-α activated binding of NFκB transcription factor to the response elements in a concentration-dependent manner in both cell lines (Fig. 4D).

### 4VP diminished the blood vessel formation of zebrafish embryos

**Figure 5:**
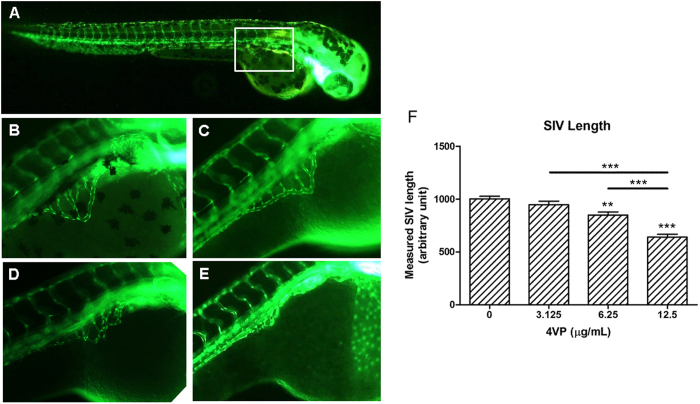
Effects of 4VP on the formation of subintestinal vessel (SIV) in zebrafish embryos. (**A**) Representative images of zebrafish embryos at 72 hours post-fertilization with normal SIV formation after incubation in vehicle. A smooth basket-like structure was enlarged and shown in (**B**). The length of SIV was assessed as described in materials and methods. (**C–E**) Upon treatment with 4VP (3.125, 6.25 or 12.5 μg/mL, respectively), the formation of SIV was impaired. (**F**) The average length of SIV in the total of 40–50 zebrafish embryos were calculated and plotted in bar chart. Data are expressed as mean + SEM. Differences among treated groups were determined by one-way ANOVA with Tukey’s post-hoc test. **p < 0.01, ***p < 0.005 as compared among groups.

Zebrafish embryos were treated with 4VP (3.125, 6.25 or 12.5 μg/mL) or vehicle or SU5416 for 72 hours. At 48 hpf, abnormal development of intersegmental vessels could be observed in the embryos treated with 4VP and SU5416 (data not shown). In order to assess the effect of 4VP on blood vessel growth quantitatively, the length of subintestinal vessel (SIV) was measured at 72 hpf^32^,^34^. The vehicle-treated control group (Fig. 5A,B) had normal vessel development, in which the SIV form as a smooth basket-like structure. As shown in Fig. 5C–E, SIV formation was blocked by 4VP in a dose-dependent manner. In the group treated with 12.5 μg/mL of 4VP, the formations of SIV were diminished. When the length of SIV was measured, results showed that the 4VP treatment (6.25–12.5 μg/mL) significantly decreased the length of SIV in zebrafish embryos (*p* < 0.01, Fig. 5F).

### Anti-angiogenic effects of 4VP in mouse Matrigel plug model

**Figure 6:**
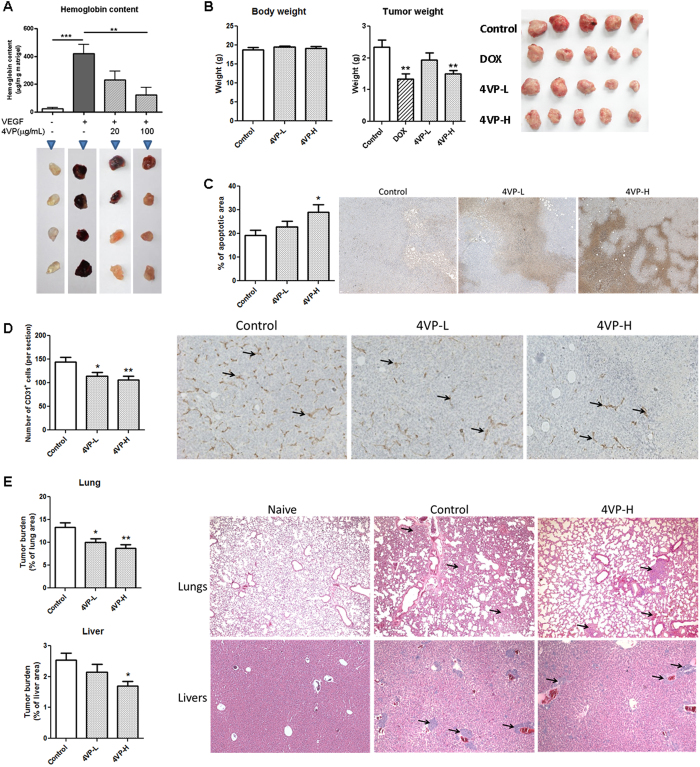
Inhibitory effects of 4VP on angiogenesis in mouse models. (**A**) Upper: Hemoglobin content of Matrigel plugs from indicated groups (n = 10-11). Lower: The representative pictures of Matrigel plugs from indicated groups at day 7 after inoculation into mice. (**B**) The body and tumor weights of 4T1 tumor-bearing mice after Control (vehicle), 4VP-L (0.2 mg/kg) or 4VP-H (2 mg/kg) treatments. (**C**) Tumor apoptotic area and (**D**) endothelial cells in the tumor sections were assessed using TUNEL assay and CD31 immunohistochemical analysis, respectively. Representative photomicrographs in (**D**) showing the endothelial cells stained with anti-CD31 antibodies in brown. The paraffin-embedded sections of the lungs and livers were photographed and used to measure tumor area and total lung or liver area. The histograms showed the tumor burden in (**E**) lungs and (**F**) livers according to the tumor area as a percentage of whole lung or liver area per group. Representative H&E-stained sections of lungs and livers from different groups with arrows in (**E**) showing the tumor nodules (Naive group: no tumor inoculated and no treatment). Data are expressed as mean + SEM. Differences between the treated and vehicle-treated control groups were determined by one-way ANOVA with Tukey’s post-hoc test. *p < 0.05, **p < 0.01, ***p < 0.005 as compared among groups.

To further verify the inhibitory effect of 4VP on angiogenesis, the *in vivo* Matrigel plug assay was performed. The plugs containing VEGF and heparin exhibited red color indicating that new blood vessels formation (angiogenesis) occurred in the plugs (Fig. 6A, lower panel). In the presence of 4VP, plugs were of light red or pale pink color indicating that less blood vessels were formed. The extent of angiogenesis was quantified by measuring the hemoglobin content in the plugs. Figure 5G histogram showed that the hemoglobin concentrations in the plugs loaded with VEGF plus 20 or 100 μg/mL 4VP (230.6 or 122.2 μg hemoglobin/mg Matrigel) were significantly lowered than those in the plugs loaded with VEGF alone (420.7 μg hemoglobin/mg Matrigel). The result is consistent with the suppression of angiogenesis by 4VP *in vitro*.

### *In vivo* anti-angiogenesis of 4VP in mouse breast tumor-bearing mice

In order to investigate the *in vivo* activities of 4VP on angiogenesis, a tumor-bearing mouse model was employed, in which mouse 4T1 breast tumor cells were injected into the mammary fat pad of female BALB/c mice. Tumor-bearing mice were intraperitoneally injected with vehicle, 4VP-L (0.2 mg/kg) or 4VP-H (2 mg/kg) for 4 weeks. During the treatment there was no observable body weight loss and the final body weights were similar in vehicle-treated or 4VP-treated mice (Fig. 6B). At the end of experiment, tumors were excised from each animal for the determination of tumor weights and the assessment of the cell death using *in situ* TUNEL assay. Tumor weights of 4VP-H treatment groups were significantly decreased by 36.3% when compared with control group, while doxorubicin (DOX, 2 μM) treatment decreased by 42.9% (Fig. 6B). The apoptotic areas in tumors were increased by 51.2% in 4VP-H treatment when compared with control group (Fig. 6C). Immunohistological analysis showed that the formation of neovasculatures stained by CD31-specific antibody in tumors (Fig. 6D). The CD31-positive cells (brown spots in photos) were found to be decreased in 4VP-treated mice in a dose-dependent manner. Hence, the administration of 4VP in tumor-bearing mice may inhibit the angiogenesis in the tumors, which then deplete the blood supply for the tumors and block the tumor growth.

Furthermore, the effects of 4VP on the metastasis of tumor cells in 4T1 tumor-bearing mice were also investigated. Lungs and livers from each mouse were removed for assessing the tumor burden. Numerous tumor nodules were found in vehicle-treated control group, while the tumor areas were decreased in 4VP-treated group in a dose-dependent manner (Fig. 6E–F). Tumor burden in lungs was found to decrease by 34.6% (lung metastasis decreased from 13.3% to 8.7%) in 4VP-H-treated group when compared with vehicle-treated group (Fig. 6E). In addition, the tumor burden of livers was also decreased in 4VP-H treatment group.

## Discussion

4-vinylphenol (4VP) naturally occurs in coffee, peanuts and wild rice^21^,^22^ and has not been found in the annual medicinal herb *Hedyotis diffusa* prior to this study. 4VP has been identified as a metabolite of styrene and the pneumotoxicity and hepatotoxicity at its high doses have been reported^22^,^26^,^27^. The cytotoxicities of 4VP and other styrene metabolites in mouse lung Clara cells have been evaluated and the LD_50_ value of 4VP was 3.5 mM^27^. Nevertheless, the biological activity of 4VP has seldom been examined. In the present study, we demonstrated for the first time that 4VP possessed anti-angiogenic effects in both tested human endothelial cells and the underlying mechanisms have also been elucidated. Furthermore, our *in vivo* studies data showed that the non-toxic dose of 4VP could reduce tumor size and blood vessel growth in tumors in breast tumor-bearing mouse model.

Our findings demonstrated that 4VP could inhibit human endothelial cell proliferation, which was assessed by thymidine incorporation assay, with IC_50_ values around 160 μM, suggesting that non-toxic low dose of 4VP may exert beneficial biological activities. New vessels formation (angiogenesis) involves multistep process such as cell proliferation, migration, tube assembly and remodeling. Suppression at any step may result in new blood vessel formation inhibition^37^. Inhibitory effects of 4VP on endothelial cell migration, wound-induced motility and tube formation suggested its potential anti-angiogenic properties.

The effects of 4VP on the ability of HUVEC and HMEC-1 to adhere to extracellular matrix (ECM) proteins have been examined in order to further investigate the possible mechanisms through which 4VP may exert their inhibitory effects on invasion and migration of endothelial cells. Treatment of 4VP for 2 hours decreased the ability of the cells to adhere to type II and IV collagen, fibronectin, vitronectin and laminin in both tested endothelial cell types (Fig. 2E). Collagen IV is important components of the extracellular matrix and plays a role in cell adhesion and motility^38^. Fibronectin has an important role in promoting endothelial cell survival and migration. It has also been shown to bind and enhance VEGF activity in mediating endothelial cell migration^39^. Vitronectin is a high molecular weight glycoprotein and known to promote cell adhesion and affect cell migration^38^. Laminin and collagen IV were suggested to be essential for endothelial differentiation in whole vascular tubes on Matrigel^40^. Hence, treatment with 4VP resulted in decreased adhesion of endothelial cells to these matrix proteins and in turn reduced the ability of migration of endothelial cells.

Vascular and extracellular matrix remodeling involve a variety of cell types and influence the angiogenic response in tumor microenvironment^3^. Degradation of the underlying basement membrane by endothelial cells of the existing blood vessels is a crucial step for initiating the formation of new capillaries. Such process requires the cooperative activity of the matrix metalloproteinases (MMPs) system and thus MMP activity is a prerequisite for angiogenesis^41^. MMP2 and MMP9 are specific for the degradation of type IV collagen. Other ECM components, such as Type I and V collagen, laminin and fibronectin are also the substrates of MMP2^42^. The activities and protein levels of active-MMP2 in both endothelial cell lines were significantly decreased after 4VP treatment, whereas the changes of MMP9 protein level were only observed in 4VP-treated HUVEC. Thus, the mRNA expressions of MMP2 and MMP9 were evaluated in HUVEC only. Inhibition of active-MMP2 and MMP9 expressions and decreased adhesion of endothelial cells to ECM by 4VP may cooperate to reduce degradation of ECM and thereby the inhibition of angiogenesis.

On the other hand, the protein and mRNA expressions of vasoactive molecule receptors (VEGFR1, VEGFR2 and Tie 2) were shown to be decreased in both 4VP-treated HUVEC and HMEC-1 endothelial cells. Although the test cell lines have been reported with distinct receptor levels and different abilities of endogenous VEGF production^43^,^44^, the suppressive effects of 4VP on receptor expressions were comparable in two cell lines. Since the amount of VEGF receptors was higher in HUVEC, the suppressive effects of 4VP on mRNA expressions have been further elucidated. Our findings suggested that 4VP could suppress the expressions of VEGF receptors in two types of endothelial cells, which have heterogeneity in the morphology, function and gene-expression profiles.

In addition to the inhibitory effects on ECM remodeling and VEGFRs expressions, based on our Western blot results (Fig. 4), 4VP was hypothesized to suppress the endothelial cell proliferation by activating p21 and inactivating ERK signaling. Our results also showed that the protein levels of cyclin B1 and D1 decreased to different extents in 4VP-treated HUVEC and HMEC-1. Meanwhile, the expression of phosphorylated ERK was suppressed in greater extent in HMEC-1 than that in HUVEC. Interestingly, apparent decreases in PI3K, pPI3K and pAKT were observed in 4VP-treated HMEC-1. Such changes of signaling molecules may be responsible for the inhibition of proliferation and survival in HMEC-1 endothelial cells. On the other hand, decreased P38 MAPK and phosphorylation of Src was observed in both 4VP-treated HUVEC and HMEC-1. The inhibition of P38 MAPK and Src signaling pathway may account for the suppressed migration^45^. The roles of 4VP in NF-κB expression and activation in human endothelial cells were elucidated in the present study. The results showed that 4VP suppressed the NFκB activation in both endothelial cell lines.

In one of our *in vivo* studies, 4VP was shown to inhibit the formation of subintestinal vessels (SIV) in zebrafish embryos *in vivo*. Zebrafish embryo model used for high-throughput screening of pro-angiogenic or anti-angiogenic agents is efficient and easy to manipulate. The embryos exhibit several features of tumor biology, such as rapidly dividing cells, apoptosis and angiogenesis^46^. We have previously demonstrated the anti-angiogenic activities of some herbal extracts or their isolated compounds in zebrafish model^32^,^47^. In the present study, the positive control SU5416 and 4VP could interfere the development of intersegmental vessels at 48 hpf; however, the quantitative analysis of SIV at 72 hpf could only be performed for 4VP-treated embryos, as the color of SU5416 masked the SIV. In the *in vivo* mouse Matrigel plug model, 4VP could decrease the hemoglobin content in Matrigel plugs loaded with VEGF in comparison with VEGF alone. These *in vivo* results substantiate the inhibitory effects of 4VP, which is able to prevent vascular endothelial cells from responding to VEGF.

Last but not least, the anti-angiogenic, anti-tumor and anti-metastasis effects of 4VP have been demonstrated in a mouse 4T1 breast tumor model, which closely mimics later stage of breast cancer in humans. After 4VP treatment, no significant difference was shown on body weights. The intraperitoneal administration of 4VP was able to decrease the tumor weights and blood vessels numbers in tumors as well as increase the cell death in tumors. The tumor weight inhibition of 4VP was comparable to positive control doxorubicin treatment. In addition, 4T1 cells are documented to be highly invasive and primary tumors typically metastasize to the lungs and livers after establishment for 2 to 3 weeks in BALB/c mice. Our data showed that 4VP treatment decreased the tumor cell metastasis to lungs and livers. Metastasis, an outgrowth of cancer cells in distant organs, is a complex cascade of events including local invasion, intravasation, extravasation and angiogenesis. Hence, the anti-angiogenic effects of 4VP may contribute to the tumor growth arrest by blocking the blood supply as well as the metastasis amelioration by preventing the new blood vessel formation. Since 4VP has been identified as a metabolite of styrene and suggested to be pneumotoxic and hepatotoxic^22^,^26^,^27^, the toxic effects were observed in mice treated with intraperitoneal injection of 50–200 mg/kg 4VP. In contrast, in the present study the tested doses were 25-fold lower (0.2 and 2 mg/kg) and no obvious change in body weight after 4-week treatment. There was no observable injury in lung sections as mentioned in previous toxicity studies.

On the other hand, 4VP was isolated from *Hedyotis diffusa* for the first time in the present study. The compound 4VP in HD aqueous extract might be converted from *p*-coumaric acid by enzymatic reaction^25^. *p*-Coumaric acid could be found in HD^48^ and its antiangiogenic effects of *p*-coumaric acid have been reported recently^49^. The *in vitro* inhibitory activities of 4VP (40 μg/mL equivalent to 0.3 mM) on tube formation and migration of endothelial cells were comparable to those of *p*-coumaric acid (1 mM). However, 4VP may possess stronger *in vivo* anti-angiogenic activities than *p*-coumaric acid because 4VP at 2 mg/kg could significantly inhibit angiogenesis in breast tumors while the effective dose of *p*-coumaric acid was 150 mg/kg^49^.

In conclusion, the anti-angiogenic effects of 4VP on HUVEC and HMEC-1 cells may act through inhibition of the PI3K/AKT signaling and MMP activation, leading to the decrease in cell proliferation and migration/invasion, respectively. 4VP may exert anti-angiogenic activity by down-regulation of VEGFR expressions. Besides, the results of *in vivo* studies using zebrafish and tumor-bearing mice suggested that the non-toxic dose of 4VP could significantly inhibit the blood vessel growth and in turn exert anti-tumor effects. These findings may provide evidence of 4VP for its potential use as an anti-angiogenic agent.

## Additional Information

**How to cite this article**: Yue, G. G.-L. *et al.* Novel PI3K/AKT targeting anti-angiogenic activities of 4-vinylphenol, a new therapeutic potential of a well-known styrene metabolite. *Sci. Rep.* **5**, 11149; doi: 10.1038/srep11149 (2015).

## Supplementary Material

Supplementary Information
