# Supplementary material for: Novel PI3K/AKT targeting anti-angiogenic activities of 4-vinylphenol, a new therapeutic potential of a well-known styrene metabolite
Source: Sci Rep. 2015 Jun 8;5:11149. doi: 10.1038/srep11149 (PMC4459151; doi:10.1038/srep11149)
Supplement: Supplementary Information [file srep11149-s1.doc]

**Novel PI3K/AKT targeting anti-angiogenic activities of 4-vinylphenol, a new therapeutic potential of a well-known styrene metabolite**

Grace Gar-Lee Yue^1,2^, Julia Kin-Ming Lee^1,2^, Hin-Fai Kwok^1,2^, Ling Cheng^1,2^, Eric Chun-Wai Wong^1,2^, Lei Jiang^1^, Hua Yu^1^, Hoi-Wing Leung^1,2^, Yuk-Lau Wong^1^, Ping-Chung Leung^1,2^, Kwok-Pui Fung^1,2,3^ and Clara Bik-San Lau^1,2,*^

^1^Institute of Chinese Medicine, The Chinese University of Hong Kong, Shatin, New Territories, Hong Kong

^2^State Key Laboratory of Phytochemistry and Plant Resources in West China (CUHK), The Chinese University of Hong Kong, Shatin, New Territories, Hong Kong

^3^School of Biomedical Sciences, The Chinese University of Hong Kong, Shatin, New Territories, Hong Kong

***Extraction and isolation of 4-vinylphenol***

The dried herb *Hedyotis diffusa* (14 kg) was extracted twice with distilled water under reflux for 1 hour. The combined aqueous extract was concentrated *in vacuo* to give a brown residue (1.26 kg). The residue was then suspended in methanol. The methanol solution was partitioned successively with dichloromethane and *n*-butanol, respectively. The dichloromethane extract (35.9 g) was subjected to silica gel column chromatography eluted with n-hexane-EtOAc (8:2, 6:4, 4:6, 2:8 v/v), EtOAc, EtOAc-methanol (8:2, v/v) and methanol to yield two fractions. Fraction 1 (2.13 g) was then further fractionated by silica gel column chromatography eluted with n-hexane-EtOAc (9:1, 8:2, 7:3, 6:4, v/v), EtOAc and methanol to obtain six further subfractions (F1-1 to F1-6). F1-2 (101.2 mg) was applied to silica gel column chromatography using n-hexane-dichloromethane (1:1, v/v) to furnish two subfractions (F1-2A and F1-2B). F1-2A was purified using Sephadex LH-20 (25-100 μm, GE Healthcare, USA) column chromatography eluted with chloroform-methanol (9:1, v/v) to afford the compound 4-vinylphenol (45 mg, 0.0036% w/w). The identification of the purified compound (4-vinylphenol) was based on the ^1^H and ^13^C NMR spectral analysis and mass spectrometry. The content of 4-vinylphenol in HD aqueous extract was determined by HPLC analysis.

***Quantification of 4VP in HD extract***

The content of 4VP in HD aqueous extract was determined by HPLC analysis after pretreatment with Oasis MAX (3 cc) SPE cartridges (Waters Co. Massachusetts, USA). Briefly, 0.5 mL of the extract (50 mg/mL in water) was passed through the cartridges after equilibrated with 1 mL methanol and 2 mL water. Cartridges were washed with 2 mL water, 2 mL of 10% methanol and 1 mL of 30% methanol in sequence. Finally cartridges were eluted with 0.5 mL methanol. The elutions were collected and 20 μL of which were injected into the HPLC for analysis. The content of 4VP in extract was expressed as the percentage of 4VP in total extract.

Separation of 4VP from the matrix was achieved by an Agilent HP1100 system (Hewlett Packard, Agilent, USA) coupled with a Beckman ODS C-18 analytical column (250 mm × 4.6 mm I.D., 5 μm) and protected with a Beckman ODS C-18 guard-column (12.5 mm × 4.6 mm I.D., 5 μm) maintained at 35 °C. The elution of analytes was performed with a mobile phase of A (Water) and B (methanol) under a gradient program of 40-70-100% B by a linear increase from 0-20-20.1 min and keeping at 100% B for 5 more minutes. The flow rate was set at 1.5 mL/min and the injection volume was 20 μL. The analytes were monitored at the UV wavelength of 258 nm.

***Tube formation assay***

Formation of capillary tube like structures by HUVEC and HMEC-1 was assessed in Matrigel-based assay as described previously (Yue et al., 2011). Briefly, a 96-well plate coated with 60 µL of Matrigel per well was allowed to solidify at 37 °C for 1 hour. Cells (1.5 × 10^4^ in 100 μL medium) were added into each well and 100 µL of medium containing 10 - 40 μg/mL of 4VP were added and incubated for different periods of time (8 hours for HUVEC and 6 hours for HMEC-1). The enclosed networks of tubes were photographed under inverted microscope (Olympus IX-71, Tokyo, Japan). The total tube lengths of the tube structure of each photograph were measured using ImageJ software (version 1.43, NIH, USA). The equation of the inhibition was:

% of inhibition= (1- (tube length treated/ tube length control)) x 100

***Cell migration assay***

To assess the cell migration ability of HUVEC and HMEC-1, a modified Boyden chamber assay was used as described previously (Yue et al., 2011). Briefly, cells (3 × 10^4^ in 100 μL medium) were added into each transwell filter chamber with 8 µm pore size (Corning, USA). At the same time, 100 µL of medium containing various concentrations of 4VP (with 1% v/v FBS) was added to the upper chambers. Five hundred microliters of medium (with 10% v/v FBS) served as chemoattractant media was added to the lower chambers. The migrated cells were quantified by manual counting in blinded fashion. The change in cell number is represented as a percentage of control.

The cell motility of HUVEC and HMEC-1 was also evaluated using scratch wound assay as described previously (Yue et al., 2011, 2013). In brief, cells (1 × 10^5^ in 1 mL medium) seeded in the wells of 24-well plate were scraped with a cross. Then the medium was changed with fresh medium with 10 - 40 μg/mL of 4VP. The cells were incubated for different time intervals (24 hours for HUVEC and 16 hours for HMEC-1) and each well was photographed. The percentages of open wound area were measured and calculated. The changes of open wound area represent the motility of cells across the scratch wound. The lower the motility of cells resulted in greater open wound area.

***Extracellular matrix cell adhesion assay***

Cell adhesion of HUVEC and HMEC-1 was assessed using Extracellular Matrix Cell Adhesion Array Kit (Chemicon, Millipore, USA). Each eight-well strip consists of wells pre-coated with 7 different human ECM proteins (collagen I, collagen II, collagen IV, fibronectin, laminin, tenascin and vitronectin) and one BSA-coated well (negative control). The assay was carried out according to the procedures recommended in the assay kit manual. In brief, the cells were added to pre-coated wells treated with 20 or 40 μg/mL of 4VP for 2 hours. After washing, attached cells were stained and the cell-bound stain was then extracted in extraction buffer. The absorbance of the stain was determined. The change in absorbance was represented as fold of untreated control (without 4VP).

***Real time-PCR analysis***

Human endothelial cells HUVEC and HMEC-1 (1 × 10^6^/mL) were seeded and incubated for 24 hours. Different concentrations (20 or 40 μg/mL) of 4VP were added to the dishes and incubated for 24 or 48 hours. After treatments, cells were harvested and washed. Total RNA was extracted, quantitated and subjected to reverse transcription as described previously (Yue et al., 2011; 2013). Reverse transcription of the 3 μg total RNA was performed in Bio-Rad iCycler (Bio-Rad, Hong Kong) using SuperScript III Reverse Transcriptase reagents according to the manufacturer’s protocols. To quantify the amount of mRNA of *MMP2, MMP9*, *Tie 2, VEGFR1* and *VEGFR2*, real time semi-quantitative PCR of cDNA samples were performed in Bio-Rad CFX96^TM^ Real-time system C1000 Thermal cycler using the iTaq Fast SYBR Green Supermix from Bio-Rad. Each 20 μL PCR sample contained 80 ng cDNA, 10 μL Supermix, RNase-free water and 1.25 μL of both the specific forward and reverse primers (10 mM). The primers were synthesized by Life Technologies (Hong Kong) and the sequences were listed in Table S1 (see below). Reactions were performed in triplicate using the following protocol: 3 min preincubation at 95 °C, followed by 40 PCR cycles at 95 °C for 3s, 60 °C for 30 s, and 72 °C for 5 s. Relative quantification was obtained by the comparative threshold cycle (*ΔΔ*Ct) method (CFX Manager Software, version 1.6, Bio-Rad). The specific gene mRNA levels were normalized relative to GAPDH mRNA level in each sample.

***Immunohistochemical analysis***

The tumor sections were also stained with anti-mouse CD31 (Dianova, Germany) antibody using an immunohistochemical method. Briefly, the paraffin-embedded slides were blocked in Peroxidased 1 blocking reagent (Biocare, USA) for 5 minutes and then washed in distilled water. The slides were deparaffinized using Diva Declocker reagent (Biocare), washed in Tris buffered saline and incubated with CD31 primary antibody in TBS/0.1% Tween 20 (1:50) for 2 hours at room temperature. After the incubation, the slides were washed and incubated with rat probe followed by horseradish peroxidase-streptavidin conjugate. The slides were washed and color was developed using Stable 3,3’-Diaminobenzidine as chromogen. Finally, sections were washed in distilled water, counterstained with Lillie-Mayer’s hematoxylin, and mounted for evaluation. Four fields of tumor sections were randomly selected, and the CD31 stained cells (in brown) was counted in each field.

**Table S1 Sources and catalogue numbers of antibodies for western blot**

| **Names of antibodies** | **Companies** | **Catalogue numbers** |
| --- | --- | --- |
| β-actin | Sigma-Aldrich | A5316 |
| AKT | Cell Signaling | #4685 |
| pAKT | Cell Signaling | #4058 |
| Cyclin B1 | BD Biosciences | 554177 |
| Cyclin D1 | BD Biosciences | 554180 |
| ERK | Cell Signaling | #4695 |
| pERK | Cell Signaling | #4377 |
| MMP2 | Cell Signaling | #4022 |
| MMP9 | Cell Signaling | #2270 |
| NFκB p65 | Cell Signaling | #3034 |
| p21 | BD Biosciences | 554228 |
| P38 | Cell Signaling | #9212 |
| pP38 | Cell Signaling | #9215 |
| PI3K | Cell Signaling | #3358 |
| pPI3K | Cell Signaling | #4228 |
| Src | Cell Signaling | #2123 |
| pSrc | Cell Signaling | #2101 |
| Tie2 | Cell Signaling | #4224 |
| VEGFR1 | Cell Signaling | #2893 |
| pVEGFR2 | Cell Signaling | #2478 |
| HRP-Goat Anti-Rabbit IgG | Life Technologies | 65-6120 |
| HRP-Goat Anti-Mouse IgG | Life Technologies | 62-6520 |

**Table S2**  Gene specific PCR primers

|  | Forward primer | Reverse primer |
| --- | --- | --- |
| *TIE2* | 5’-TTGAAGTGGAGAGAAGGTCTG-3’ | 5’-GTTGACTCTAGCTCGGACCAC-3’ |
| *VEGFR1* | 5’-CCAGCAGCGAA AGCTTTGCG-3’ | 5’-GAGGCCATCGCTGCA CTCA-3’ |
| *VEGFR2* | 5’-GCAGGGGACAGAGGGACTTG-3’ | 5'-GAGGCCATCGCTGCACTCA-3' |
| *MMP2* | 5’-CAAAAACAAGAAGACATACAT-3’ | 5'-GCTTCCAAACTTCACGCTC-3′ |
| *MMP9* | 5’-TTGACAGCGACAAGAAGTGG-3’ | 5’-GCCATTCACGTCGTCCTTAT-3’ |
| *GAPDH* | 5’-CGAGATCCCTCCAAAATCAA-3’ | 5’-TTCACACCCATGGACGAACAT-3’ |

**Table S3 ^13^C and ^1^H NMR data for compound 4VP (measured in CDCl_3_)**

| **Position** | **δC** | **δH (*J* in Hz )** | **COSY** | **HMBC** |
| --- | --- | --- | --- | --- |
| 1 | 155.795 ( C ) |  |  | 7.268 , 6.783 |
| 2 | 115.385 ( CH ) | 6.783 d (2H, J=8.0) | 7.268 | 7.268, 6.640 |
| 3 | 127.528 ( CH ) | 7.268 d (2H, J=8.0) | 6.783 | 6.783, 6.640, |
| 4 | 130.271 ( C ) |  |  | 6.783 , 6.640 , 5.583 |
| 5 | 136.237 ( CH ) | 6.640 dd (1H, J=10.5, 17.5) | 5.583  5.100 | 7.268 , 5.583 , 5.100 |
| 6 | 111.304 ( CH2) | 5.583 d (1H, J=17.5)  5.100 d (1H, J=10.5) | 6.640 | 6.640 |
